# Special Issue “Third Edition: Advances in Molecular Simulation”

**DOI:** 10.3390/ijms25052709

**Published:** 2024-02-27

**Authors:** Małgorzata Borówko

**Affiliations:** Department of Theoretical Chemistry, Institute of Chemical Sciences, Faculty of Chemistry, Maria Curie-Skłodowska University, 20-031 Lublin, Poland; malgorzata.borowko@mail.umcs.pl

Molecular simulation is one of the fastest growing fields in science. This is connected with the rapid increase of computer efficiency and with the appearance of new, sophisticated simulation methods enabling the modeling of complex systems while seamlessly bridging different length and time scales. Now, it is possible to obtain accurate values for the macroscopic properties of various compounds and materials starting from atomistic quantum mechanical calculations. These parameters can be used to simulate physical and chemical phenomena, as well as biochemical processes occurring in living cells. Modern research requires an interdisciplinary approach and covers several issues, including classical, “pencil” theories, experiments, and just computer simulations. Only the combination of these different views and methodologies guarantees successful work in the long run.

Today, the focus of research in the field is much more on the mechanism of processes than on the system structure. Scientists ask questions like “How it is working?” or “How does this happen?”, instead of “What does this look like?”. Questions about mechanisms are generally difficult to answer using experimental techniques. Therefore, theory is a necessary complement to experiments.

The theoretical methods used today are based on both quantum mechanics and classical physics. In the quantum chemical approach, electrons and atomic nuclei are the particles of interest. However, in the classical model atoms, groups of atoms are the particles that are considered. This means that classical models contain much fewer degrees of freedom and they are consequently evaluated much faster using a computer. Furthermore, the equations used to describe the classical particles are much simpler and this also contributes to speeding up the computer calculation.

Skillfully combining these two approaches, quantum mechanics and classical physics, is a real art that requires knowledge and experience. How important this problem is for the development of computational methods was proven by the fact that The Royal Swedish Academy of Sciences decided to award the 2013 Nobel Prize in Chemistry to Martin Karplus, Michael Levitt, and Arieh Warshel, for the “development of multiscale models for complex chemical systems”. The laureates proposed methods to develop models that describe part of a system using the first principle, quantum chemical models for a central part of the system, and how to link this part to a surrounding, all of which is treated within the framework of classical physics. The most important achievement was showing how these two regions could interact in a physically realistic way. Such hybrid models are currently used in many theoretical studies and are involved in computer simulations.

In general, two methods for theoretical studies can be used: “pure” theories and molecular simulations. Among these theories are advanced quantum methods, sophisticated statistical–-thermodynamic approaches, and simple phenomenological calculations. Just twenty years ago, the appreciation and significance of theoretical research in natural sciences were at very high levels, and many new ideas came from the theory of polymers, fluids, phase transitions, critical phenomena, etc. Theory is still very important to formulate a proper system of notions and new ideas in science. However, the situation has changed dramatically; now, computer simulation is becoming the dominant tool in natural sciences.

Molecular simulations contain a great variety of special procedures that allow us to mimic real systems and their evolution over time. We observe the constant and rapid development of simulation methods. The current state of the art in this field is the subject of numerous, continuously published reviews [1,2,3]. The most popular simulation techniques are different versions of the molecular dynamics method and Monte Carlo method [1,3]. Molecular simulations can also be performed using cellular automata [4] or genetic algorithms [5], and can be supported by the use of machine learning [6,7], various expert systems [8], and artificial intelligence [9].

The key problem in simulation is to assume proper interactions between all species. For this purpose, numerous force fields were developed to estimate the interaction potentials between atoms or coarse-grained particles. A force field provides functions for potential energies and a set of their best-fitted parameters. Such potentials are fit to the results of quantum mechanical calculations and, usually, to certain experimental measurements.

In molecular simulation, the trajectory of the system in the configuration space can be generated stochastically, based on the laws of mechanics, or both. The rules for accepting these states are based on changes in the energy of the system. There have been impressive advances in simulation techniques over recent years. Simulations can be performed in different statistical ensembles, including the extended ensembles used in multicanonical simulations or the parallel tempering method. A lot of enhanced sampling techniques [10] and different methods for the analysis of simulation data [11] have recently been proposed.

Currently, molecular dynamics is likely the most popular simulation method. This is mainly due to the availability of many ready-made software packages that provide all the major molecular dynamics codes, along with a set of accompanying programs to facilitate writing simulation scripts. Molecular dynamics simulations are a mature technique that can be used effectively to understand the mechanism of complex processes and macromolecular structure-to-function relationships.

Although molecular dynamics simulation is used in many fields, the most spectacular development is in biochemistry and molecular biology [12,13]. Modern simulations enable deep insight into the structure of macromolecules and allow the calculation of many parameters describing their behavior. This changes the usual paradigm of structural bioinformatics from the study of single structures to the analysis of conformational ensembles. Molecular dynamics simulations capture the behavior of proteins and other biomolecules in full atomic detail and at very fine temporal resolutions. The method has turned out to be very valuable in deciphering the functional mechanisms of these molecules, in exploring the structural basis for disease, and in the design of small molecules, peptides, and proteins. The applications of these methods span different topics, for example, molecular docking, allosteric regulation, structure refinement, drug discovery, and many others [12]. The information collected from simulations may motivate further experimental work. All these achievements have meant that molecular simulations are finding more and more applications in medicine [14,15], pharmacy [16], and even food science [17].

In the future, we can expect that molecular simulations will be increasingly used to model new structures in nanotechnology [1,18,19,20]. We are observing the rapid development of this field, caused by the emergence of increasingly spectacular applications of nanomaterials. One of the “hot topics” is the use of molecular simulations to model the energy storage process [21].

Molecular simulations are becoming easier and more accessible to users who do not need to have deeper knowledge of the underlying procedures. Attention should be paid to the correct analysis of the simulation results and their validation.

We are probably approaching another breakthrough in simulation techniques, related to the use of artificial intelligence. This tool is so powerful that it is difficult to predict the consequences of its use.

The main purpose of this Special Issue is to show a wide range of applications of molecular simulation. We present here articles that deal with three topics: (i) the modeling of processes occurring at interfaces, (ii) the determination of properties of modern materials, and (iii) the investigation of the structure of complex macromolecules.

Three different processes are considered, namely the behavior of hybrid particles (Janus particles and hairy particles) at fluid–fluid interfaces, the growth of graphene on Cu(111) surfaces via chemical vapor deposition, and the transport of nonpolar molecules through a nanotube. The properties of polymer-bonded explosives are also explored using molecular dynamics. Moreover, the applications of computer simulations for the determination of the structure and properties of aggregates of dye, as well as the compounds of major cat allergies, are reported. The diversity of the research presented here confirms the importance of molecular simulations for the development of various, often very disparate fields of science.

I believe that all the articles collected here contribute to progressing molecular simulations of important physical, chemical, and biological processes.

Finally, I am pleased to thank the people who worked on this Special Issue: the authors, reviewers, and editors.
